# Establishment of thromboelastography reference intervals by indirect method and relevant factor analyses

**DOI:** 10.1002/jcla.23224

**Published:** 2020-01-31

**Authors:** Daye Cheng, Xiaoying Li, Shuo Zhao, Yiwen Hao

**Affiliations:** ^1^ Transfusion Department First Affiliated Hospital of China Medical University Shenyang China

**Keywords:** indirect method, reference interval, thromboelastography, transfusion

## Abstract

Thromboelastography (TEG) as a global coagulation test has been continuously developed for many decades in either research or clinical practice. The versatility of TEG test leads to difficulty in standardization and result interpretation. Reference intervals (RIs) of TEG may be one of the most controversial factors that influence its wide applications. RIs establishment with the traditional method is time‐consuming and laborious as well as beyond general laboratory capability. Indirect method using stored data and with statistical calculation and small cost is emerging as an alternative approach for RIs determination. Gender, age, or both affect RIs and must be taken into account before RIs estimation. The present study retrospectively collected a total of 930 TEG results as subjects and established RIs with indirect method for Kaolin‐activated TEG, including the parameters of *R*, *K*, αAngle, MA, and CI. Furthermore, gender, age, and gender‐dependent age subsets analyses were performed to determine their effects on RIs of TEG. In this study, we found that TEG parameters showed more hypercoagulability in female than male, most of the measured TEG variables were significantly associated with aging, but only in male statistical significance was found among different age stratification and 60‐year‐old could be considered as cutting point to differentiate coagulation ability in male. In addition, RIs of TEG were estimated by indirect method suitably and verified to be valid in our study. Finally, the RIs of TEG by indirect method were basically significantly different to the RIs recommended by manufacturer, but the consistent percentage is relatively high in the most of measured parameters. In conclusion, it is suggestive that the indirect method for RIs establishment is feasible, but relevant factors, such as gender and age, specifically gender‐dependent age effect, should be considered before RIs determinations.

## 1 INTRODUCTION

Thromboelastography (TEG) was introduced 70 years ago and was designed for assessing the overall coagulation process in vitro.^1^ With the generally used parameters (*R*, *K*, αAngle, MA, LY30, and CI, as described in Table S1), TEG is able to produce a graphical trace representing the main steps occurring in coagulation process and provide global information about hemostatic status.^2^ Compared with conventional coagulation examinations, such as prothrombin time (PT), activated partial thromboplastin time (APTT), and fibrinogen concentration, TEG is able to monitor coagulation in nearly real‐time and with less turnaround time.^3^, ^4^ Thus, TEG has been increasingly applied in massive bleeding surgery, such as cardiac operation^5^, ^6^, ^7^ and liver transplantation,^8^ to guide blood transfusion. In emergent settings, TEG also plays critical roles in diagnosis of coagulopathy for traumatic patients and is helpful in making therapeutic strategies for transfusions.^9^ Furthermore, TEG platelet mapping is arising more interesting in monitoring platelet reactivity on treatment to direct a tailored antiplatelet therapy recently.^10^, ^11^

Although TEG is a useful tool in directing transfusion and monitoring antiplatelet management, a number of limitations do not support its wide application. So far, most of the TEG devices can only be performed manually without automation. In addition, a variety of types of sample, including whole blood, citrated anticoagulant sample, and plasma sample as well as platelet‐rich plasma sample can be adopted in TEG test. Furthermore, different initiators for activating coagulation process, such as Kaolin or tissue factors can be also applied in TEG.^2^ Therefore, the versatile ability of TEG increases the variability and leads to difficulty in standardization of this test, which also causes an absence of universally suitable reference intervals (RIs) for TEG.

Reference intervals are of critical value for clinicians to interpret laboratory results and subsequently make diagnosis and intervention decision. As to TEG test, establishment of RIs for measured parameters and derived variables mentioned above is necessary before its clinical application, as recommended by manufacture and technical reports.^2^, ^12^, ^13^, ^14^, ^15^, ^16^, ^17^ RIs of TEG have been rarely extensively studied, with a few reports on new born,^18^, ^19^ pediatric patients,^20^ healthy children,^21^ and pregnant women.^22^ Considering demographic effect on TEG, RIs have been reported for adult in a few studies.^23^, ^24^, ^25^

Traditional method for RIs determination generally includes several consecutive steps, which mainly involves predefining criteria for “healthy participant,” calculating sufficient number needed for calculating robust RIs under statistical requirement, recruiting qualified participants, sampling, and testing. This method is historically classical and also called direct method, but is time‐consuming and expensive. In addition, it is not easy to define “healthy participant” criteria.^26^ The underlying diseases may influence greatly on RIs establishment due to relatively small number of participants recruited. For example, 120 subjects are the minimum requirement according to the official recommendation.^27^ Considering the difficulties of the direct method in RIs determination, it is not practical for all the laboratories to produce their RIs for targeted serving population.^26^ Thus, many laboratories choose either RIs recommended by manufactures or those transferred from other laboratories, even without validation of their laboratories serving population.^26^

Another method for RIs determination, which is an indirect method, is highly encouraged by the International Federation of Clinical Chemistry (IFCC) for laboratories to use in establishing RIs.^26^, ^28^ Indirect method is relatively simple, less expensive, and time‐saving. This method takes advantage of Laboratory Information System (LIS) to retrospectively collect a larger number of data from routine testing records and uses appropriate statistical tools based on data type, distribution, and related factors to produce RIs. Furthermore, this method is especially suitable to such environment when extreme limitations are defined, such as newborn, pregnant, or advanced age subjects involving in the targeted population, which leads to extreme difficulty to recruit sufficient number of subjects with direct method. Thus, indirect method for RIs has been increasingly used for establishing RIs in various medical fields.^29^, ^30^, ^31^

In this study, our aims were to produce Kaolin‐activated TEG RIs with indirect method and compare them with those from manufacturer's recommendation; to validate the RIs derived from our studied population; and to analyze relevant factors potentially effecting TEG parameters including gender, age, and gender‐dependent age effect on TEG values.

## 2 METHODS

### 2.1 Date collection for indirect method

The TEG result records including parameters of *R*, *K*, αAngle, MA, Ly30, and CI of the participants from Health Examination Center of our hospital (First Affiliated Hospital of China Medical University, Shenyang, China) were collected and included in this study. Giving TEG data was not recorded by LIS before August, 2017, and data collection time was from August, 2017, to July, 2019. Among collected TEG data, only the results of TEG plain cup test with Kaolin as initiator were used for RIs determination, while for platelet mapping or other types of examinations were excluded. In addition, data of repeated examinations, which may possibly be related to potential risk of coagulating diseases, were also excluded.

### 2.2 TEG assay procedure

All TEG tests were performed by using Thrombelastography5000 (Haemoscope Corporation). One milliliter of citrated whole blood was placed into a 1% kaolin vial (Medtel; for Hemoscope Corp.), which was then inverted five times to ensure appropriate activation of the sample. After activation by kaolin, 20 μL of 0.2 mol/L calcium chloride was added into each TEG cup and then 340 μL of whole blood was loaded into the cup. All the tests were performed at 37°C, and the assay was run for at least 60 minutes until completion of the measurement of clot lysis at 30 minutes. The electric internal quality control (*e*‐test) was performed at least three times a day. As recommended by manufacturer's instructions, all the tests were performed within 3 hours after sample collection. Test results were recorded by the TEG computer software TEG^®^V4 (Haemoscope Corporation) for later analyses. According to manufacturer's instructions of Thrombelastography5000, reference value ranges of the TEG parameters are as follows: *R* (reaction time, normal range: 5‐10 minutes); *K* (*K* time, normal range: 1‐3 minutes); α (alpha angle, normal range: 53° to 72°); MA (maximum amplitude, normal range: 50‐70 mm), CI (coagulation index, normal range: −3 to 3) and LY30 (lysis at 30 minutes, normal range: 0%‐8%). And detailed information was described in the technical report.^2^

### 2.3 Quality control

In order to ensure the accuracy of the collected data, a special investigator (Zhao Shuo) was responsible for the daily quality control with *e*‐test and chemical control once a month, and who was also in charge of data collection. Stability of TEG devices was vital to the quality of TEG data, so we examined stability of TEG devices by grouping and comparing data from each of the eight channels. Moreover, TEG test might vary during data collecting time span, therefore, the collected data were divided into two groups in terms of data collecting year to examine differences.

### 2.4 Statistical method

All the analyses were performed by SPSS, version 20.0 for Windows (SPSS Inc). And Microsoft Excel for Windows, version 2010, and GraphPad 8.0.2 were used as the auxiliary software. Data distribution regarding normality was assessed by checking the histogram and using the Anderson‐Darling test. The Tukey method was used to identify and remove outliers.^32^, ^33^ In detail, the 25th percentile and 75th percentile were calculated as Q1 (1st quartile) and Q3 (3rd quartile), and interquartile ranges (IQRs) were equal to Q3 minus Q1. The outliers were determined as long as the data lied outsides of the range of Q1−1.5*IQR to Q3+1.5*IQR. For stability examination, data from each of the eight channels were grouped and compared by one‐way analysis of variance (ANOVA) or nonparametric tests based on the data distribution. Moreover, data were divided into two groups in terms of data collecting year and compared using t test or nonparametric tests according to the data distribution.

### 2.5 Establishment and validation of RIs

After treating of data, the remained data were used for establishment of RIs of TEG parameters through using the Reference Value Advisor software, which was described in detail by Geffre et al.^34^ The Reference Value Advisor software can be used in Microsoft Excel to calculate reference limits through different methods. For different series of data, Reference Value Advisor uses a nonparametric method to calculate reference limits and 90% confidence intervals [CI]. Furthermore, the established RIs with indirect method were validated with 20 subjects with routine order of TEG examination in our Health Examination Center. In detail, the 20 subjects were selected with randomized method without consideration of gender and age and were tested with routine batch of examination randomly. Two or less results out of 20 falling outside of the derived RIs would be considered as valid with the criteria of a 95% probability, as recommended by IFCC.^26^, ^27^

## 3 RESULTS

**Figure 1:**
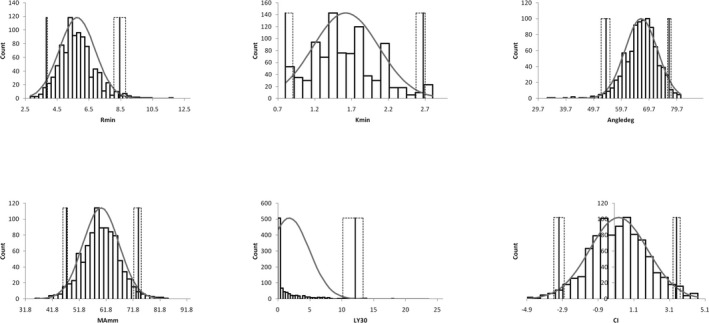
Distributions of all parameters measured by thromboelastography (TEG) are presented by histograms. Reference intervals for all TEG parameters with 90% confidence interval are also shown. The solid gray line represents the upper and the lower limit of the reference interval. The black dotted line indicates the 90% confidence interval. The gray line indicates the Gaussian curve. Abbreviations: *R* min = reaction time of minutes, *K* min = clotting time of minutes, Angledeg = alpha angle with unit of degree, MA mm = maximum amplitude with unit of millimeter, LY30 = lysis at 30 min after MA, CI = coagulation index

The data of 1126 participants were collected. After screening subjects and deleting outlier, a total of 930 (584 male, 62.8%) participants were included. MA showed normal distributions in total and male group (*P* = .100 and .227, respectively), and CI showed normal distribution in total and female group (*P* = .117 and .217, respectively). The other parameters in different groups were not normally distributed but visually close to Gaussian distribution, except of the most skewed LY30; in addition, all the detected parameters between male and female were compared and significant differences were found with female exhibiting more hypercoagulable than male in the overall parameters, detailed information is shown in Table 1 and Figure 1.

**Table 1:** Data distributions of all thromboelastography parameters and differences between male and female subsets

Parameters	Group	N (%)	Mean	Median SD	Minimum	Maximum	*P* a	*P* b
*R* (min)	Total	930	5.75	5.70 ± 1.13	2.80	11.80	<.010	<.010
	Male	584 (62.8)	5.86	5.80 ± 1.10	3.00	11.80	<.010	
	Female	346 (37.2)	5.58	5.50 ± 1.15	2.80	9.20	<.010	
*K* (min)	Total	930	1.62	1.60 ± 0.44	0.80	2.80	<.010	<.010
	Male	584 (62.8)	1.72	1.70 ± 0.45	0.80	2.80	<.010	
	Female	346 (37.2)	1.46	1.40 ± 0.39	0.80	2.80	<.010	
αAngle (degree)	Total	930	66.87	67.30 ± 5.90	33.00	81.20	<.010	<.010
	Male	584 (62.8)	65.68	66.20 ± 5.94	33.00	81.20	<.010	
	Female	346 (37.2)	68.89	69.50 ± 5.27	49.20	81.10	<.010	
MA (mm)	Total	930	59.87	59.80 ± 6.71	35.40	84.80	.100	<.010
	Male	584 (62.8)	59.02	58.70 ± 6.81	41.60	81.70	.227	
	Female	346 (37.2)	61.31	61.30 ± 6.29	35.40	84.80	.010	
LY30 (%)	Total	930	1.77	0.20 ± 3.15	0.00	23.60	<.010	<.010
	Male	584 (62.8)	4.33	3.00 ± 4.08	0.10	23.60	<.010	
	Female	346 (37.2)	4.48	3.25 ± 4.30	0.10	23.60	<.010	
CI	Total	930	0.25	0.20 ± 1.63	−5.40	4.70	.117	<.010
	Male	584 (62.8)	−0.05	−0.10 ± 1.62	−5.40	4.70	.009	
	Female	346 (37.2)	0.75	0.90 ± 1.53	−4.40	4.50	.217	

Note

Anderson‐Darling test was used for testing all parameters data distribution.

^a^ *P* < .05 indicates an abnormal distribution.

^b^ *P* < .05 indicates significant difference between M and F group with respective parameters.

**Figure 2:**
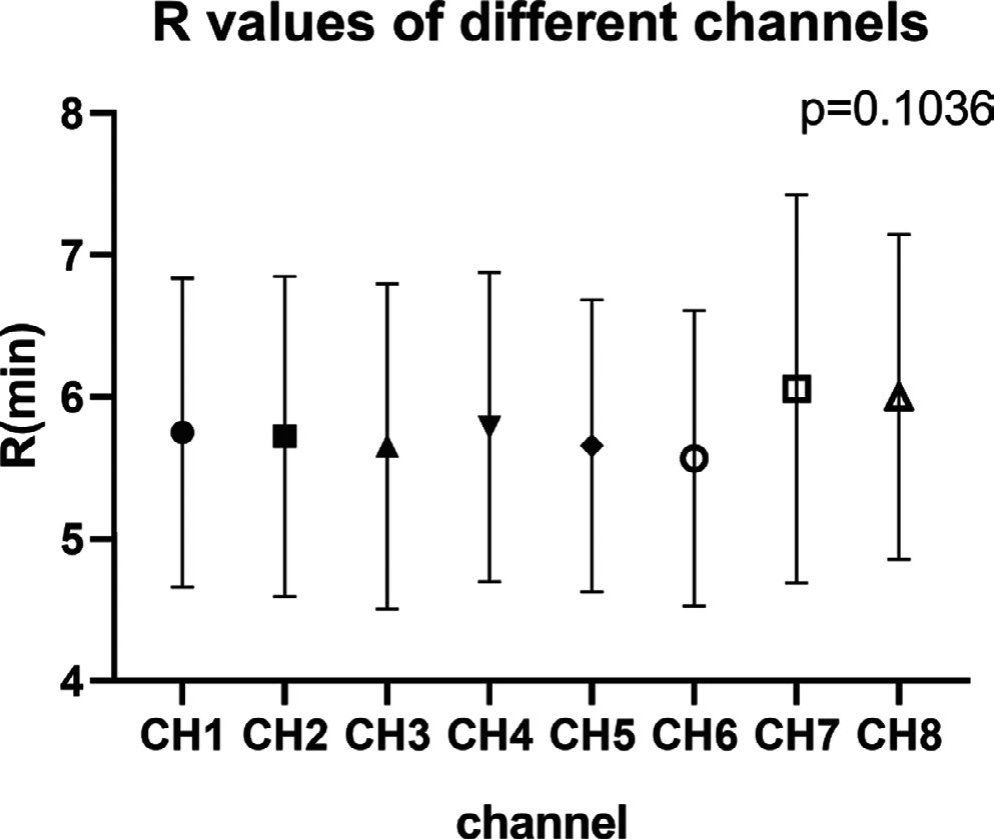
*R* parameter values measured by eight channels from four thromboelastography devices; CH means channel and the number followed means respective channels used for examination. *R* min = reaction time of minutes

For device stability assessment during the period of data collection, *R* was chosen as a representative parameter and the results of *R* from each of the eight channels of four TEG devices were compared, no significant differences were found (*P* = .1036), as shown in Figure 2. Furthermore, environmental effect on TEG tests during the two consecutive data collecting years was tested by *R* comparison results of the groups divided by data collecting year, and similarly, no significant differences were seen (data not shown). As no differences were found for various device channels and no significant variation over the two consecutive years, data were combined for analyzing thereafter.

**Figure 3:**
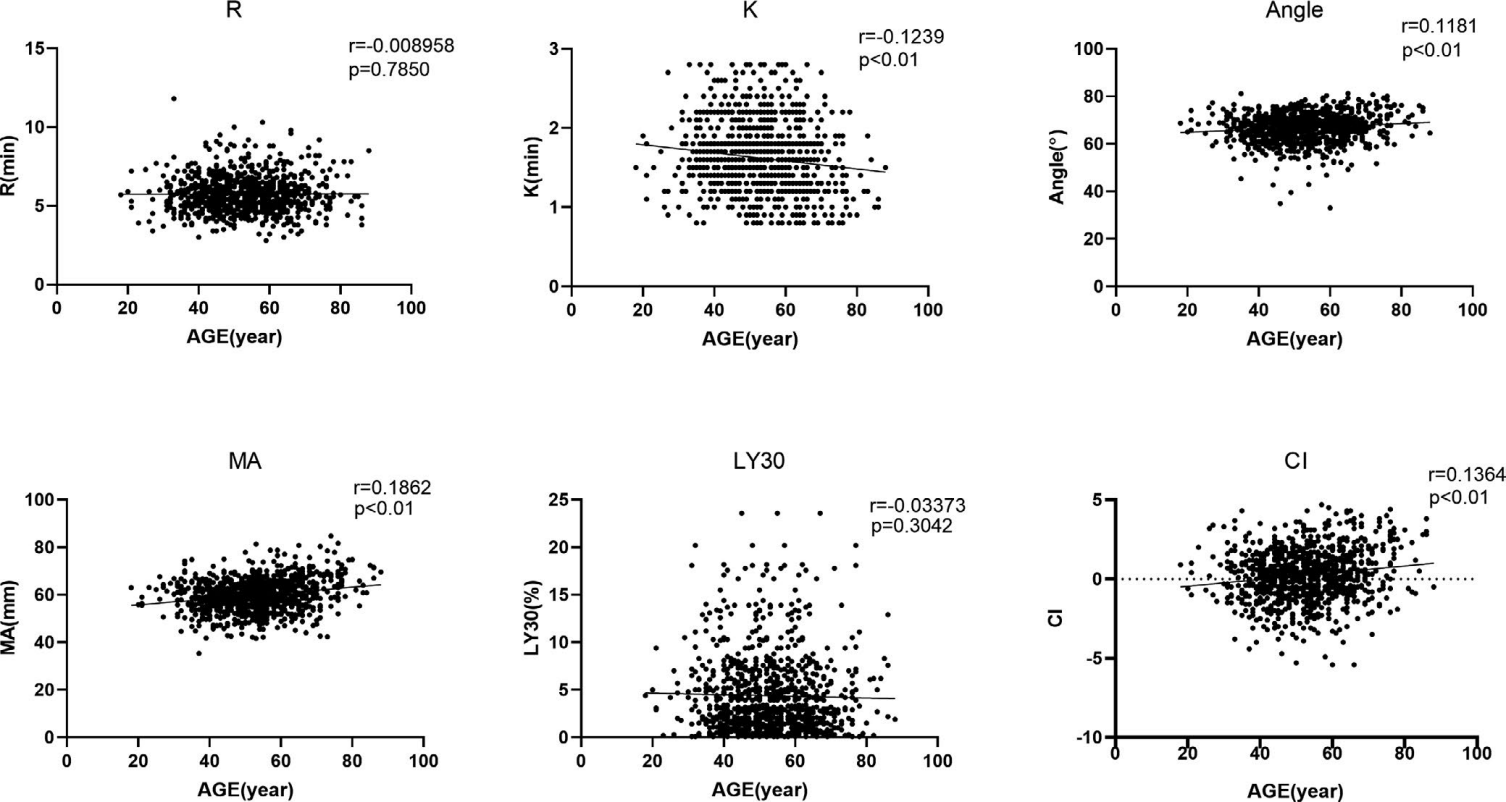
Correlations of all thromboelastography parameters measured with age (y) in total group. *R* = reaction time of minutes, *K* = clotting time of minutes, Angle = alpha angle with unit of degree, MA = maximum amplitude with unit of millimeter, LY30 = lysis at 30 min after MA, CI = coagulation index

**Figure 4:**
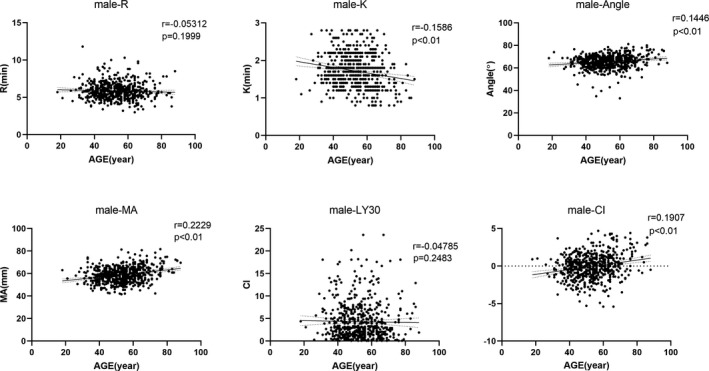
Correlations of all thromboelastography parameters measured with age (y) in male group. *R* = reaction time of minutes, *K* = clotting time of minutes, Angle = alpha angle with unit of degree, MA = maximum amplitude with unit of millimeter, LY30 = lysis at 30 min after MA, CI = coagulation index; Male‐parameter indicates respective parameter in male population

**Figure 5:**
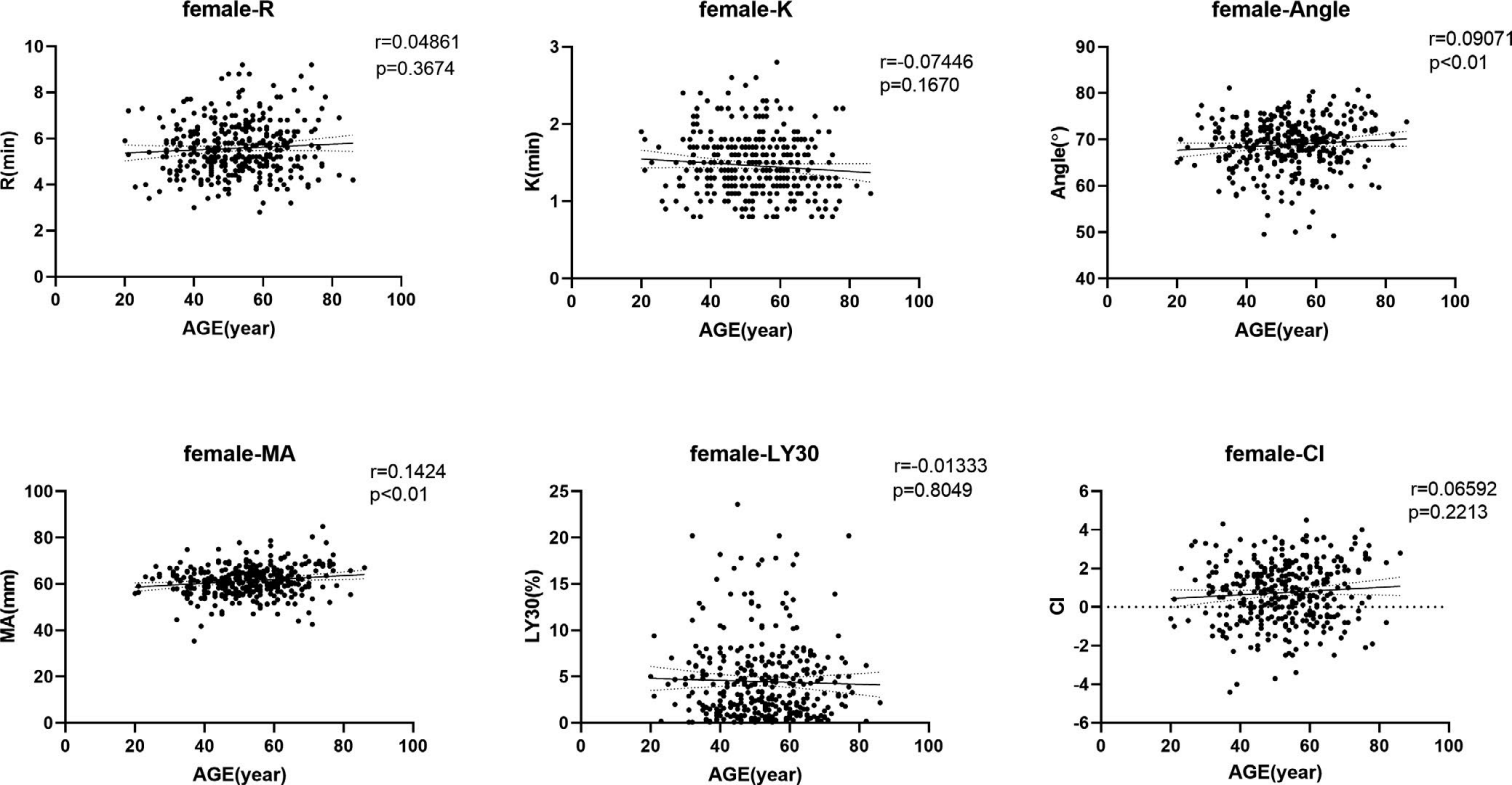
Correlations of all thromboelastography parameters measured with age (y) in female group. *R* = reaction time of minutes, *K* = clotting time of minutes, Angle = alpha angle with unit of degree, MA = maximum amplitude with unit of millimeter, LY30 = lysis at 30 min after MA, CI = coagulation index; female‐parameter indicates respective parameter in female population

Considering that age would potentially have influence TEG results as gender did, all the measured parameters were tested by linear correlation with age. In total group, as illustrated in Figure 3, αAngle, MA, CI showed significantly positive correlation with age increasing (*r* = .1181, .1862, .1364, respectively, and *P* < .01 for all); and negative correlations were found in *R*, *K*, and LY30 with age advancing, but only *K* showed statistical significance (*r *= −.1239, *P* < .01). To address whether different gender would be confounded in age effects on TEG results, the total group was stratified to male and female subgroups and then linear correlation tests between all parameters and age were further performed. As illustrated in Figures 4 and 5, more prominent correlations were found in male compared with female subset. To be detailed in male group, *K* (*r* = −.1586, *P* < .01), αAngle (*r* = .1446, *P* < .01), MA (*r* = .2229, *P* < .01), and CI (*r* = .1907, *P* < .01) showed significant correlation with age; but in female group, only αAngle (*r* = .0907, *P* < .01) and MA (*r* = .1424, *P* < .01) showed significant correlation with age. We did not find any significant correlation between *R*, LY30, and age in total, male and female group (all *P* > .05).

Taking into account whether different effects on TEG parameters by age is gender specific, further stratification analyses based on age (≤40, 41‐50, 51‐60, and >60 years) for total, male, and female were performed. As shown in Table 2, it was surprisingly found that all parameters of TEG for female participants showed no significant difference among the four age‐stratified groups. While analyses among male subgroups for various age exhibited significantly different for all parameters, except for *R* values (*P* = .865). Thus, further comparisons among different age subgroups of male were performed for differentiation. It was demonstrated that only the subgroup of participants >60 years was significantly different with all the other age‐divided subgroups in all TEG parameters, except of *R* parameter.

**Table 2:** Comparison for age group in total, male, and female participants

	<40	41‐50	51‐60	>60	*P* value
Total					
N (%)	138 (14.8)	260 (28.0)	292 (31.4)	240 (25.8)	
*R* (min)	5.73 ± 1.16	5.76 ± 1.12	5.78 ± 1.06	5.72 ± 1.19	.865
*K* (min)	1.63 ± 0.45	1.69 ± 0.43	1.64 ± 0.44	1.52 ± 0.45	<.001
αAngle (deg)	66.90 ± 5.42	65.96 ± 5.78	66.64 ± 5.98	68.13 ± 6.03	.001
MA (mm)	58.77 ± 6.63	58.66 ± 5.76	59.58 ± 6.58	62.16 ± 7.32	<.001
LY30 (%)	2.11 ± 3.16	2.09 ± 3.56	1.85 ± 3.20	1.14 ± 2.20	.015
CI	0.13 ± 1.61	0.00 ± 1.51	0.17 ± 1.61	0.68 ± 1.72	<.001
Male group					
N (%)	75 (54.3%)	170 (65.4%)	189 (64.7%)	150 (62.5%)	
*R* (min)	5.94 ± 1.18	5.91 ± 1.13	5.86 ± 0.98	5.75 ± 1.17	.865
*K* (min)	1.75 ± 0.45	1.81 ± 0.41	1.72 ± 0.43	1.59 ± 0.50	<.001
αAngle (deg)	65.40 ± 5.30	64.68 ± 5.68	65.46 ± 5.91	67.23 ± 6.30	.001
MA (mm)	57.70 ± 6.60	57.62 ± 5.63	58.45 ± 6.70	61.96 ± 7.43	<.001
LY30 (%)	2.47 ± 3.93	1.74 ± 3.34	1.73 ± 3.17	0.94 ± 1.75	.004
CI	−2.93 ± 1.37	−0.37 ± 1.46	−0.14 ± 1.57	0.54 ± 1.82	<.001
Female group					
N (%)	63 (45.7)	90 (34.6)	103 (35.3)	90 (37.5)	
*R* (min), mean (SD)	5.50 ± 1.09	5.47 ± 1.07	5.64 ± 1.18	5.68 ± 1.23	.729
*K* (min), mean (SD)	1.49 ± 0.41	1.48 ± 0.38	1.47 ± 0.41	1.41 ± 0.35	.659
αAngle (deg), mean (SD)	68.70 ± 5.04	68.39 ± 5.17	68.80 ± 5.51	69.63 ± 5.26	.490
MA (mm), mean (SD)	60.03 ± 6.50	60.63 ± 5.52	61.65 ± 5.82	62.48 ± 7.17	.097
LY30 (%), mean (SD)	1.68 ± 2.76	2.75 ± 3.87	2.06 ± 3.26	1.46 ± 2.76	.069
CI, mean (SD)	0.62 ± 1.73	0.70 ± 1.37	0.74 ± 1.54	0.90 ± 1.52	.767

Note

All values were presented mean ± SD. *R* represents reaction time, the time from initiation to initial fibrin formation of 2 mm amplitude; *K* represents the time taken for the amplitude to increase from 2 to 20 mm; αAngle (degree) means The angle between the midline and the tangent to the main body of thromboelastography (TEG) trace; MA represents the amplitude at the widest point of TEG trace; LY30 (%) represents the percentage reduction in amplitude 30 min after MA is reached; CI means coagulation index.

Thus, the RIs were estimated by indirect method and established for total, male, and female, which is presented with the RIs recommended by manufacturer in Table 3; the RIs of TEG by indirect method were further given 90%CI to the upper and lower limits. Because age influenced significantly on RIs of TEG only in male subjects and 60 year was statistically identified as cutting point for age stratification, the respective RIs for male >60 years and ≤60 was calculated separately, as presented in Table 4.

**Table 3:** Reference intervals of different population

N (%)	Total 930 (100)		Male 584 (62.8)		Female 346 (37.2)		Manufacturer	
Parameter (90%CI)	Lower limit	Upper limit	Lower limit	Upper limit	Lower limit	Upper limit	Lower limit	Upper limit
*R* (min)	3.8 (3.80‐3.90)	8.45 (8.07‐8.80)	3.9 (3.80‐4.16)	8.54 (8.10‐8.90)	3.67 (3.20‐3.80)	8.23 (7.80‐8.80)	5	10
							N/A	N/A
*K* (mm)	0.8 (0.80‐0.90)	2.67 (2.57‐2.70)	0.9 (0.80‐0.90)	2.7 (2.70‐2.80)	0.8 (0.80‐0.90)	2.3 (2.20‐2.50)	1	3
							N/A	N/A
αAngle (degree)	54.23 (52.41‐55.54)	76.92 (76.37‐77.67)	53.41 (52.01‐55.00)	76.2 (75.54‐77.38)	57.24 (51.10‐58.80)	77.7 (76.70‐79.30)	53	72
							N/A	N/A
MA (mm)	47 (45.69‐47.43)	73.75 (72.10‐74.90)	46.2 (45.35‐47.30)	73.69 (71.60‐74.90)	47.07 (44.00‐50.80)	74.16 (71.60‐77.90)	50	70
							N/A	N/A
Ly30 (%)	0 (0.00‐0.00)	12.11 (10.09‐13.37)	0.2 (0.10‐0.20)	15.95 (13.90‐17.78)	0 (0.00‐0.10)	12.73 (10.50‐14.40)	0	8
							N/A	N/A
CI	−3.1 (−3.40‐2.80)	3.5 (3.30‐3.70)	−3.24 (−3.61 to −3.04)	3.6 (3.10‐3.80)	−2.33 (−3.40 to −1.90)	3.5 (3.30‐3.70)	−3	3
							N/A	N/A

Note

*R* represents reaction time, the time from initiation to initial fibrin formation of 2 mm amplitude; *K* represents the time taken for the amplitude to increase from 2 to 20 mm; αAngle (degree) means the angle between the midline and the tangent to the main body of thromboelastography (TEG) trace; MA represents the amplitude at the widest point of TEG trace; LY30 (%) represents the percentage reduction in amplitude 30 min after MA is reached; CI means coagulation index.

**Table 4:** Reference intervals of thromboelastography (TEG) for male with different age

Parameter	>60 y		≤60 y	
	Lower limit (90%CI)	Upper limit (90%CI)	Lower limit (90%CI)	Upper limit (90%CI)
*R* (min)	3.63 (3.00‐3.80)	8.57 (7.80‐9.80)	4.20 (4.10‐4.30)	8.63 (8.01‐9.00)
*K* (mm)	0.80 (0.80‐0.80)	2.62 (2.40‐2.80)	1.00 (0.90‐1.10)	2.80 (2.70‐2.80)
αAngle (degree)	52.2 (51.10‐57.30)	78.55 (77.40‐81.20)	54.05 (45.35‐55.30)	74.31 (73.40‐75.30)
MA (mm)	48.42 (42.40‐50.20)	77.42 (74.70‐81.70)	45.79 (44.64‐47.00)	70.10 (68.84‐72.51)
Ly30 (%)	0.00 (0.00‐0.10)	7.15 (5.50‐9.40)	0.00 (0.00‐0.00)	13.13 (9.95‐14.39)
CI	−3.42 (−5.40 to −2.20)	4.22 (3.80‐4.40)	−3.21 (−3.80 to −3.01)	2.70 (2.13‐3.10)

Note

*R* represents reaction time, the time from initiation to initial fibrin formation of 2 mm amplitude; *K* represents the time taken for the amplitude to increase from 2 to 20 mm; αAngle (degree) means The angle between the midline and the tangent to the main body of TEG trace; MA represents the amplitude at the widest point of TEG trace; LY30 (%) represents the percentage reduction in amplitude 30 min after MA is reached; CI means coagulation index.

**Figure 6:**
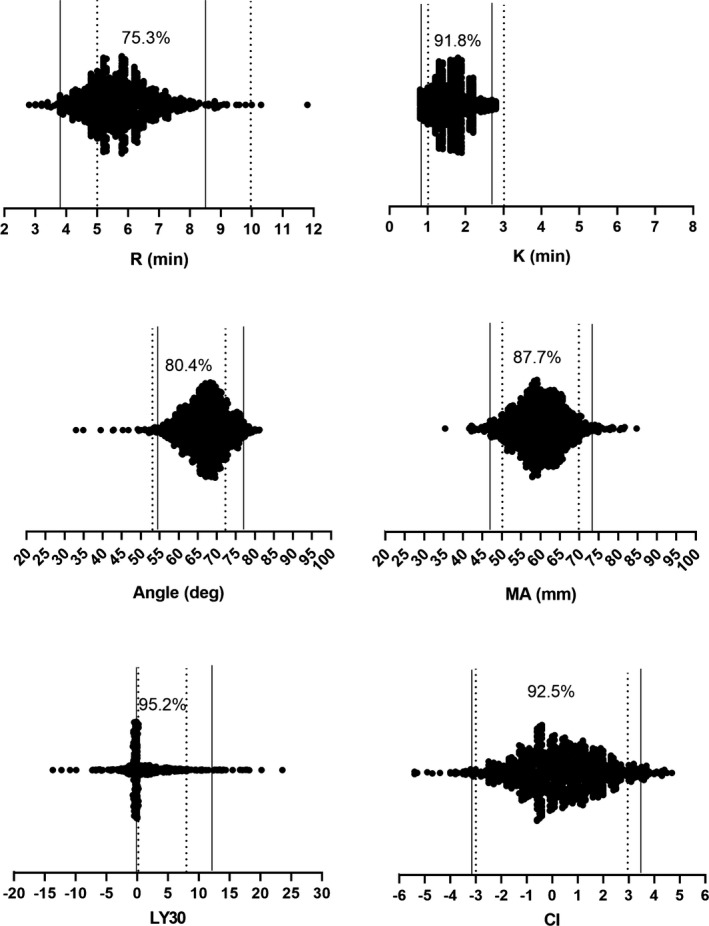
Consistencies of RIs of thromboelastography by indirect method and RIs by manufacturer. *R* min = reaction time of minutes, *R* min = reaction time of minutes, *K* min = clotting time of minutes, Angledeg = alpha angle with unit of degree, MA mm = maximum amplitude with unit of millimeter, LY30 = lysis at 30 min after MA, CI = coagulation index. The solid straight lines indicate RIs by indirect method with lower limit on the left and upper limit on the right; the doted straight lines indicate RIs by manufacturer with lower limit on the left and upper limit on the right

Further, the RIs determined in our study were compared with the RIs recommended by manufacturer. Except of *K* parameters, significant difference was demonstrated between the two RIs (by indirect method and by manufacturer) in all other variables (*K*: *P* = .225; other variables: *P* < .001) as indicated in Table 5. But the percentage of consistency between the two RIs was relative high (75.3%, 91.8%, 80.4%, 87.7%, 95.2%, and 92.5% for *R*, *K*, αAngle, MA, LY30, and CI, respectively), as shown in Figure 6.

**Table 5:** Comparisons between reference intervals of indirect method and manufacturer

Parameters	Iri	Mri			
		Above (N)	Within	Below	*P* value
*R*	Above	2	21	0	.000
	Within	0	700	192	
	Below	0	0	15	
*K*	Above	0	23	0	.255
	Within	0	854	53	
	Below	0	0	0	
αAngle	Above	23	0	0	.000
	Within	136	748	0	
	Below	0	6	17	
MA	Above	23	0	0	.000
	Within	29	816	40	
	Below	0	0	22	
LY30	Above	23	0	0	.000
	Within	22	885	0	
	Below	0	0	0	
CI	Above	22	0	0	.000
	Within	23	860	4	
	Below	0	0	21	

Abbreviations: Iri, reference intervals with indirect method; Mri, reference intervals recommended by manufacturer.

**Figure 7:**
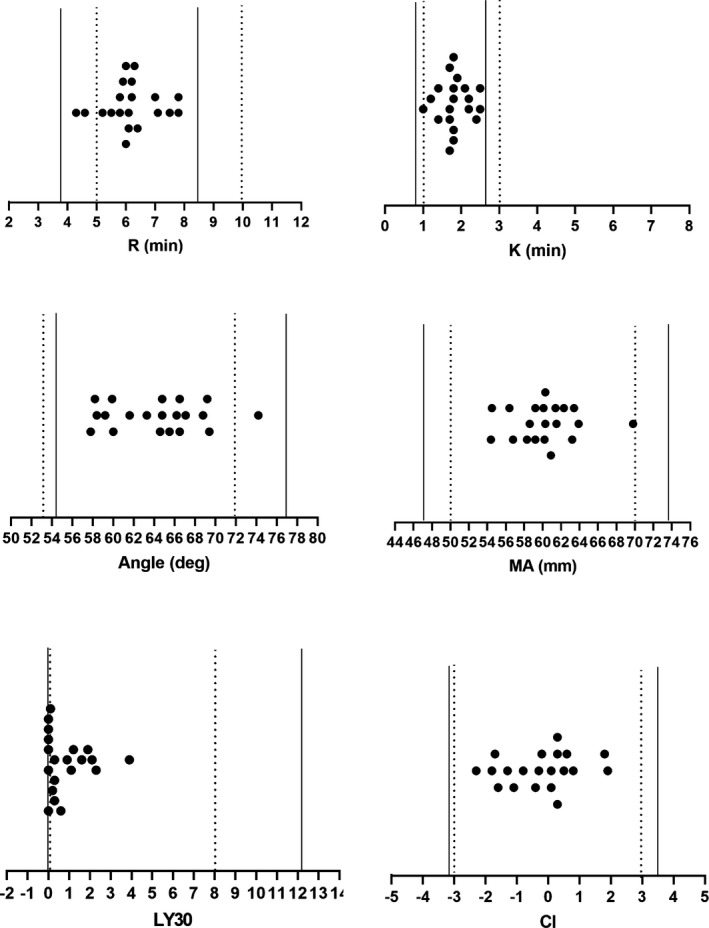
Validations of RIs of thromboelastography by indirect method. *R* min = reaction time of minutes, *K* min = clotting time of minutes, Angledeg = alpha angle with unit of degree, MA mm = maximum amplitude with unit of millimeter, LY30 = lysis at 30 min after MA, CI = coagulation index. The solid straight lines indicate RIs by indirect method with lower limit on the left and upper limit on the right; the doted straight lines indicate RIs by manufacturer with lower limit on the left and upper limit on the right

Finally, in order to validate the RIs established by the indirect method, we randomly selected 20 routine subjects with TEG examination in our Health Examination Center, and the results were illustrated in Figure 7. We found all the values used for validation were within the RIs by indirect method; but two of the *R* values and one of the Angle values were outside of the recommended RIs by manufacturer, which meant the criteria of a 95% probability established in this study was considered valid.

## 4 DISCUSSION

Coagulation is balanced between pro‐coagulant and anticoagulant factors in physical condition; otherwise either abnormal bleeding or clotting may possibly occur. It is critical to obtain coagulation information before therapeutic management being made. Thus, tests with the ability to provide timely and overall knowledge about patient coagulation status have been earning more and more attention. For the past decades, TEG is emerging as a test of detecting coagulation with overall information and short turnaround time, as compared with traditional coagulation tests such as PT, APTT, and fibrinogen concentration.^3^, ^4^, ^23^

Thromboelastography was initially described as a technique for assessing blood coagulation in 1948^1^ and has been improved continually in the past half a century. The theory and detailed techniques are elaborated by Othman and Kaur.^2^ With the generally referred parameters *R*, *K*, αAngle, MA, LY30, and CI (detailed description is in Table S1 and Figure S1) measured by TEG device, the overall coagulation process is visually provided by a continuous tracing graph transformed from the above‐mentioned parameters. According to the characteristics of the graph and parameter values, physicians can make clinical treating regimen.^6^ Thereby, TEG has been gradually applied in a number of clinical settings, especially those associated with massive bleeding, such as cardiac surgery,^5^, ^7^ liver operation,^6^, ^8^ and traumatic therapy,^35^, ^36^ mainly for guiding blood transfusion.^9^

Although TEG has the ability to direct transfusion practice and improve the outcome of clinical management,^6^, ^9^ a number of limitations prevent it from wide application. As described by several reports^2^, ^9^ that the different testing methods and various procedures can be adopted by TEG tests, which leads to difficulty in standardization. In addition, most of the TEG devices are not automatic in operation so far. Furthermore, the most important one is the absence of uniform RIs of TEG parameters, which is of great value and indispensable for clinicians to interpret TEG results.

The RIs are defined as a range of values with an upper limit and a lower limit, within which a certain possibility of the test results from targeted population would fall. Traditionally, RIs are established by following classical steps, including predefining criteria of “healthy subjects” for targeted population, enrolling participants, collecting consent forms and specimens, and testing as well as finally statistically calculating.^26^ RIs can be affected by many factors, such as ethnicity, methodology, and environmental factors.^26^ As proposed by the Clinical and Laboratory Standards Institute (CLSI) C28‐A3 document, a sufficient number of subjects are more than 120 for robust RIs establishment. One of the challenging problem to address is that the underlying diseases are difficult to be differentiated from the healthy ones and can seriously affect RIs in total, because of the relative small number of enrolled subjects as required by traditional method.^26^, ^27^It is not easy for any individual laboratory to screen and select sufficient number participants qualified for predefined criteria and successfully implement the following steps, either because of the so‐called healthy definition for enrollment or the additionally laborious testing and consumption. Thus, it is not uncommon for laboratories to use RIs directly from others or equipment manufacturer's recommendation, which may not be suitable to their own serving population and mislead the result interpretations. Recently, an alternative approach for RIs establishment, with using stored data of routine examination in LIS was recommended, namely “indirect method”.^26^, ^28^ Indirect method takes advantage of huge amount of stored data and applies sophisticated statistical tools to determine RIs. By this mean, laboratories can be relatively easy to achieve their own RIs.^26^ We sought to use this indirect method to estimate RIs of TEG parameters and analyzed related influential factors on TEG values.

In this study, we recruited 1126 pieces of TEG results from our Health Examination Center. These population are basically symptom‐free “healthy” individuals, and with the main purpose of routine health care, which would markedly reduce the effect of diseased or underlying diseased, so‐called contaminated subjects.^26^, ^28^ After screening for extreme data with visual check by distribution plot and deleting outliers by the Tukey method, a total of 930 (584 males, 62.8%) subjects remained for RIs establishment. The total distributions of retrieved data are visually close to normal except of LY30, as shown in Figure 1. Further analyses of stratified groups of gender in detail with all the parameters included, as described in Table 1, showed that most of the data were not statistically normal distribution except of MA for the total and male group, and CI for the total and female, with LY30 appeared most skewed. Thus, nonparametric method was taken to estimate RIs with the 2.5th percentile for lower limit and the 97.5th percentile for upper limit.

As recommended by the previously published reports^26^, ^28^ that measurable stability during the data collecting period must be taking into account before further analyses by using indirect method, we divided our subjects to analyze difference according to the two consecutive collecting years. As expected, no significant difference was found. In addition, to justify if different channels of TEG devices applied in this study would produce variations, data records from each of the eight channels were compared and similarly no significant difference were seen (*P* = .1036). Thus, data of the two consecutive collecting years and eight channels were combined for further analyses.

It was suggested that gender and age would influence on TEG values, we performed comparisons of the results of all measured TEG parameters by stratification based on gender, age, and gender‐specific age.^23^, ^24^, ^25^, ^37^ In our population, female presented statistically more hypercoagulablity than male population in all parameters, which was well consistent with the study that also included Chinese participants^25^; but there was no agreement with the study of Subramanian et al,^23^ which showed no significant difference in all TEG parameters between male and female with only MA showing higher in female but none of statistical significance being found; in the other studies on the RIs of TEG, they reported the similar higher coagulation ability in female as our findings, but with the exception of *R* value being absent of statistical difference between genders.^24^, ^37^ These discrepancies might be possibly explained by different device used by Subramanian et al^23^ and different ethnic population enrolled by others.^24^, ^37^

Another critical factor influencing on RIs of TEG is age. Aging is generally being linked with the risk of hypercoagulation because of the higher incidence of coagulation‐related diseases.^38^, ^39^ In the present study, TEG parameters of *K*, αAngle, MA, and CI except of *R* and LY30 were significantly correlated with aging in total and male subjects and exhibited a tendency toward hypercoagulability. But in female, only MA and αAngle showed significant correlation with aging. Thus, the main variable *R*, as an indicator to reflect plasma enzymatic factor activity, was inherently stable as age advancing. Our findings were well in line with the report of Roeloffzen et al.^37^ They demonstrated none of significant difference between age >50 and age <50 in *R* parameters in either male or female group. Furthermore, as most strikingly affected parameter by aging in our study, MA was associated with aging (*r* = .2229, *P* < .01), and which was also the most heavily correlated one in their study (*r* = .47).^37^ While in another study, age was reported not associated with hypercoagulability in both gender,^24^ that may be the cause of a small number of subjects used in their study. In our studied subjects, the coagulation status of female being less affected by aging, which was assumedly supported by the findings of other studies reporting that the incidence rate of venous thromboembolism (VTE) showed different patterns and male had significantly higher risk of VTE after 60 years old.^40^, ^41^ That gives the assumption that female continues but gets used to stably more hypercoagulabe status with less significant effect by aging compared with male counterpart, while male is increasingly hypercoagulabe with age advancing, which leads to more occurrence of hypercoagulation‐related disease.

As aging affected the TEG parameters except of *R* with statistical significance only in male in our study, we sought to divide the male participants into different age groups to differentiate which age group was independent from others. Four age‐matched groups were established according to 10 years span, that is, <40 years old, 40–50, 50–60, and above 60 years old. Promisingly, only the group of older than 60 years was significantly different to others for male (*P* < .01) in the overall TEG parameters except of *R* (*P* = .865). Thus, based on all the subjects enrolled in present study and according to analyses for gender, age, and stratified analyses for gender‐specific age, we established Kaolin‐activated RIs of TEG separately, that is, RIs for total, male, female, and for male with 60 years old as cutting point. Additionally, comparison between the RIs established by our study with indirect method and the RIs proposed by manufacturer was conducted. Similar to those reported by others,^23^, ^24^, ^25^ the RIs from manufacturer's recommendation was basically not in agreement with those determined by our study in all parameters except of *K*, but diagnostic specificity was relative high with consistent percentage of 75.3, 91.8, 80.4, 87.7, 95.2, and 92.5 for *R*, *K*, αAngle, MA, LY30, and CI, respectively. Nevertheless, it still supports the recommendation of manufacture that each laboratory should determine its own RIs of TEG before adopting this test in clinical application. Finally, according to recommendation of IFCC,^26^ we validated the RIs established by our study with 20 randomly selected samples. The results indicated that the derived RIs in the current study were valid and the indirect method for RIs with regard to TEG parameters is suitable and applicable.

Although the current study did find a number of factors influencing on TEG parameter values and established RIs for the total and subgroups with indirect method, limitations could not be ignored. First, we used relatively small number of samples for stratification analyses and RIs determination by indirect method; but the number of 930 is enough for a nonparametric method for robust establishment of RIs. Secondly, we excluded outliers without further analyses of the extreme values, which might lead to lose of useful information. Finally, we used data from routine work rather than subjects being selected with predefined criteria of healthy person as required by traditional method for RIs establishment, which might cause potential diseased related subject inclusion and affect results; but the reasonable explanation is that subjects from hospital data confer similar detecting environment in practice and is more suitable for RIs determination.^29^

To the best of our knowledge, our study firstly takes the indirect method to establish RIs for Kaolin‐activated TEG parameters of *R*, *K*, αAngle, MA, LY30, and CI. Indirect method is relatively easy and less expensive and laborious, which allows most of the laboratories to establish their own RIs for TEG as long as sufficient numbers of results are stored in LIS. Furthermore, based on the results of our study, female is more hypercoagulable than male, and the older people is more hypercoagulable than the younger in male, but female is less affected during aging process; finally, 60 years old can be considered as the cutting point to differentiate coagulation in male population, but the physical mechanisms to address the different patterns of coagulation ability for different gender and age are warranted further investigations.

## CONFLICT OF INTEREST

All authors declare no conflicts of interest in this work.
