# Supplementary figures and images for: Establishment of thromboelastography reference intervals by indirect method and relevant factor analyses
Source: J Clin Lab Anal. 2020 Jan 31;34(6):e23224. doi: 10.1002/jcla.23224 (PMC7307360; doi:10.1002/jcla.23224)

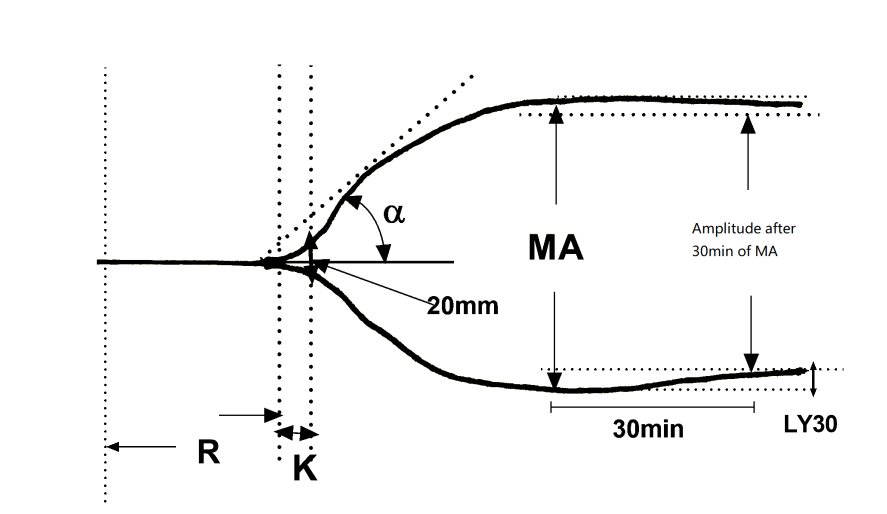

Supplement: Supplementary file 1 [file JCLA-34-e23224-s001.tif]
