# Supplementary material for: Establishment of thromboelastography reference intervals by indirect method and relevant factor analyses
Source: J Clin Lab Anal. 2020 Jan 31;34(6):e23224. doi: 10.1002/jcla.23224 (PMC7307360; doi:10.1002/jcla.23224)
Supplement: Supplementary file 2 [file JCLA-34-e23224-s002.docx]

Table S1. TEG parameters and indication

| Parameter | Description | Indication |
| --- | --- | --- |
| R(min) | Reaction time, the time from initiation to initial fibrin formation of 2mm amplitude | Initiation phase of enzymatic factor activity |
| K(min) | The time taken for the amplitude to increase from 2 mm to 20 mm | Rate of clot development |
| αAngle (degree) | The angle between the midline and the tangent to the main body of TEG trace |  |
| MA(mm) | The amplitude at the widest point of TEG trace | Maximum clot strength; reflecting contribution of platelet and fibrinogen/fibrin interaction to clot strength |
| LY30(%) | The percentage reduction in amplitude 30 min after MA is reached | Fibrinolysis activity, derived from percentage decrease in clot strength 30 min after MA is reached |
| CI | Coagulation index | Resulting from all coagulation interactions |
